# Enzymatic hydrolysis of sorghum straw using native cellulase produced by *T*. *reesei* NCIM 992 under solid state fermentation using rice straw

**DOI:** 10.1007/s13205-011-0024-6

**Published:** 2011-09-20

**Authors:** A. Vimala Rodhe, L. Sateesh, J. Sridevi, B. Venkateswarlu, L. Venkateswar Rao

**Affiliations:** 1 Department of Microbiology, UCS, Osmania University, Hyderabad, India; 2 CRIDA, Hyderabad, India

**Keywords:** Cellulase, Enzyme hydrolysis, Rice straw, Sorghum straw, *T. reesei* NCIM 992

## Abstract

Cellulose is a major constituent of renewable lignocellulosic waste available in large quantities and is considered the most important reservoir of carbon for the production of glucose, for alternative fuel and as a chemical feedstock. Over the past decade, the emphasis has been on the enzymatic hydrolysis of cellulose to glucose and the efficiency of which depends on source of cellulosic substrate, its composition, structure, pretreatment process, and reactor design. In the present study, efforts were made to produce cellulase enzyme using rice straw. The produced enzyme was used for the hydrolysis of selected lignocellulosic substrate, i.e., sorghum straw. When rice straw was used as a substrate for cellulase production under solid state fermentation, the highest enzyme activity obtained was 30.7 FPU/gds, using *T. reesei* NCIM 992. 25 FPU/g of cellulase was added to differently treated (native, alkali treated, alkali treated followed by 3% acid treated and alkali treated followed by 3 and 5% acid treated) sorghum straw and hydrolysis was carried out at 50 °C for 60 h. 42.5% hydrolysis was obtained after 36 h of incubation. Optimization of enzyme loading, substrate concentration, temperature, time and buffer yielded a maximum of 546.00 ± 0.55 mg/g sugars (54.60 ± 0.44 g/l) with an improved hydrolysis efficiency of 70 ± 0.45%. The enzymatic hydrolyzate can be used for fermentation of ethanol by yeasts.

## Introduction

The increasing concerns about the depletion, shortage of fossil fuels and air pollution caused by incomplete combustion of fossil fuels have led to specific focus on the production of cellulosic bioethanol from renewable lignocellulosic substrates such as wheat straw, rice straw, sugarcane bagasse, etc. (Sun and Cheng 2002).

Lignocellulose is considered as an attractive feedstock for the production of fuel ethanol, because of its availability in large quantities at low cost (Cheng et al. 2008) and they are composed of lignin and sugars polymerized to cellulose and hemicellulose that can be liberated by hydrolysis (acid/enzyme) and subsequently fermented to ethanol by microorganisms (Mussatto and Roberto 2004).

Rice straw is one of the most abundant lignocellulosic waste by-products worldwide and has an estimation of global production of 600–900 million tons per year (Karimi et al. 2006). Andhra Pradesh is an agricultural-based state of India. The major crops grown are rice, groundnut, maize, sugarcane and sorghum (Reddy and Sanjana 2003).

The annual production of sorghum straw in Andhra Pradesh is 764.4 tones. A total ethanol production of 0.335 GL could be achieved on the fullest utilization of the crop residue (Kim and Dale 2005). Sorghum is drought resistant and need only limited water conditions to grow. Due to its high biomass and carbohydrate contents (Mehmood et al. 2009) it is most promising for ethanol production. In our analysis sorghum straw was found to contain significant amounts of holocellulose (63%) making this substrate suitable for bioethanol production.

Regardless of the type of cellulosic feedstock, the cost and hydrolytic efficiency of enzymes are major factors that restrict the commercialization of the biomass bioconversion processes (Galbe and Zacchi 2002). Development of an economical process for cellulase production is hindered because of the high costs of substrate (pure cellulose).

Cellulase enzyme is used to break up cellulose into glucose or other oligosaccharide compounds (Acharya et al. 2008). The cellulase system in fungi comprises three hydrolytic enzymes acting synergistically (Lynd et al. 2002), endo-1,4-β-d-glucanase [carboxymethyl cellulase (EC.3.2.1.4)], which cleaves β-linkage randomly in the amorphous parts of cellulose; exo-1,4-β-d-glucanase [cellobiohydrolase (EC.3.2.1.91)], which hydrolyzes cellobiose from either non-reducing or reducing end, generally from the crystalline parts of cellulose; β-glucosidase [cellobiase (EC.3.2.1.21)], releases glucose from cellobiose and short chain cellooligosaccharides (Wilson 2009).

Commercial cellulase enzymes could be used to convert lignocellulose into reducing sugars (Wen et al. 2005). The cost of commercial cellulase enzyme is very high; hence, this process is non-economical (Von Sivers and Zacchi 1995). The enzyme production cost accounts for approximately 40% (Spano 1978) of the total cost of bioethanol production and connected to the productivity of microbial strain and the final activity of enzyme in the fermentation broth (Himmel et al. 1999). The cost of raw material is the limiting factor in developing an economic process for cellulase production (Reczey et al. 1996). The economy of cellulase production could be improved by the use of cheap cellulosic substrates for enzyme production (Lynd et al. 2002).

Enzyme complexes for the degradation of lignocelluloses have been produced by solid state fermentation (SSF) on various agricultural residues such as rice straw, wheat bran, corn stover, sugarcane bagasse, pomace, corncobs, etc. (Soccol and Vandenberghe 2003), using host-specific fungi for best results (Nigam and Singh 1996).

SSF is successfully used over submerged fermentation for large scale production of fungal metabolites as it resembles the natural habitat for filamentous fungi to grow and produce the fermented products (Pandey 2003). SSF, a highly attractive and alternative process needs to be exploited for generation of cellulolytic enzymes with the use of cheaply available lignocellulosic residues and low input of process engineering. SSF is preferred due to low capital investment and lower operating cost (Rodriguez Couto and Sanroman 2005), higher product yield, better product quality, cheaper product recovery and cheaper technology (Oguntimein et al. 1992).

Among several microorganisms evaluated so far, organisms belonging to the genera of *Trichoderma* and *Aspergillus* are known to be potential cellulase producers (Kumar et al. 2008). Currently, most commercial cellulases, including β-glucosidases, are produced by *Trichoderma* species and *Aspergillus* species (Cherry and Fidantsef 2003).

Enzymatic hydrolysis of cellulose is carried out by highly specific cellulase enzymes. A mixture of endoglucanases, exoglucanases, β-glucosidases and cellobiohydrolases is commonly used (Mansfield et al. 1999).

Making cellulose accessible to the enzymes is an essential factor to increase the rate of hydrolysis (Philippids and Smith 1995). Therefore, chemical pretreatment, usually alkaline pretreatment, is necessary before enzymatic hydrolysis. Chemical pretreatment not only removes lignin but also acts as a swelling agent, which enhances surface area of the substrate accessible for enzymatic action (Kim et al. 2008).

The enzyme hydrolysis is usually conducted at mild conditions (pH 4.8 and temperature 45–50 °C) and does not have a corrosion problem (Duff and Murray 1996). Moreover, it is possible to obtain hydrolysis of close to 100% by enzymatic hydrolysis, whereas it is difficult to achieve such high yield with acid hydrolysis. Several inhibitory compounds are formed during acid hydrolysis and this problem is not so severe for enzymatic hydrolysis (Lee et al. 1999).

The factors that affect the enzymatic hydrolysis of cellulose include substrates, cellulase activity and reaction conditions (temperature, pH, etc.). To improve the yield and rate of the enzymatic hydrolysis, optimizing the hydrolysis process and enhancing cellulase activity need to be focused (Cantwell et al. 1988).

During the enzymatic hydrolysis, cellulose is depolymerized by the cellulases to monomeric reducing sugars that can be fermented by yeasts or bacteria to ethanol.

In the present study, cellulases were produced on the alkali pretreated rice straw with *T. reesei* NCIM 992, and subsequent hydrolysis of the sorghum straw, a lignocellulosic substrate with the cellulase produced was investigated. The hydrolytic potential, in terms of total reducing sugar yields obtained with the produced cellulase, was evaluated and the sugars obtained can be further employed in bioethanol production.

## Materials and methods

### Microorganism

*T. reesei* NCIM 992 was procured from National Collection of Industrial Microorganism (NCIM), National Chemical Laboratory, Pune, India and maintained on potato dextrose agar (PDA) agar at 4 °C for subsequent use as inoculum.

### Fungus cultivation for spore production and inoculum preparation

The fungal culture was grown on PDA slants and the spores were harvested aseptically from 5-day-old PDA slants. Sterile distilled water containing 0.1% (w/v) Tween-80 (Smits et al. 1996) was added to each fungal slant and vortexed. Spore count was measured with haemocytometer and adjusted to 2 × 10^6^ spores/ml by adjustment of optical density.

### Substrate preparation and sterilization

Rice straw was obtained from the outskirts of Hyderabad, pulverized to obtain a particle size of 2–3 mm. The substrate was washed with tap water and dried in oven at 50 °C. The dried and powdered rice straw was soaked in 1% KOH for 4 h at room temperature. After treatment, excess alkali was decanted and the substrate was washed with distilled water till it reached neutral pH and dried overnight at 50 °C.

### Medium composition

The growth medium used in SSF process consisted of mineral salts and trace elements (g/l): urea, 0.3; NH_4_SO_4_, 1.4; CaCl_2_·2H_2_O, 0.4; KH_2_PO_4_, 2; MgSO_4_·7H_2_O, 0.3; peptone, 1; Tween-80, 0.2; FeSO_4_·7H_2_O, 0.005; MnSO_4_·7H_2_O, 0.016; ZnSO_4_·7H_2_O, 0.014; CoCl_2_·6H_2_O, 0.2; PSAC, 10; agar, 17.5; Triton-X 100, 1 ml; pH-5.3 (Mandels and Andreotti 1978). 1% lactose was used as an inducer (Xu et al. 2008). The medium was used to adjust initial moisture content (80%) and autoclaved at 121 °C for 10 min.

### Enzyme production under SSF

Different lignocellulosic substrates such as rice straw, sorghum straw, wheat straw, saccharum, corn cobs, etc., were initially screened for their suitability for cellulase enzyme production by *T. reesei* NCIM 992, under solid substrate fermentation. Rice straw was found to be a suitable solid substrate for higher yields of cellulase and hence in the present study, fermentation was carried out under SSF using rice straw as substrate. Solid substrate (10 g) was mixed with nutrient supplement (pH 4.5) in 250 ml flask to obtain a 1–2 cm layer of mixture without free liquid. The flasks were sterilized by autoclaving, cooled, shaken thoroughly to break the mass.

### Inoculation process

Spore suspension (2 × 10^6^ ml^−1^) was transferred at 2% (v/w) per flask. The contents in the flasks were mixed thoroughly to ensure uniform distribution of the inoculum.

### Growth and enzyme production

The growth of *T. reesei* NCIM 992 and enzyme production was carried out at 28 °C under static conditions for 6 days.

### Sampling process

The substrate was mixed for 5 min prior to sampling process. Sampling was done for every 24 h. The sample was used to determine the activity of the enzyme produced.

### Enzyme extraction

The enzyme was extracted by a simple contact method (Krishna and Chandrasekaran 1996). The fermented samples were shaken (150 rpm) with 0.05 M citrate buffer (pH 4.8) by applying substrate:buffer (S:L 1:20) concentration for 1 h and filtered through Whatman No. 1 filter paper. The filtrates were centrifuged for 20 min at 4 °C to remove spores of the organism and the supernatant of crude enzyme extract was used for enzyme assay.

### Determination of enzyme activity

Cellulase activity (FPA) was analyzed on filter paper, according to Ghose (1987). One unit of enzyme corresponds to the amount of enzyme necessary to form 1 μmol of glucose per ml per minute. The reducing sugars were measured by the dinitrosalicylic acid (DNS) method according to Miller (1959).

### Optimization of cellulase production

Factors such as amount of substrate, moisture content, concentration of inducer (lactose), incubation time and temperature that affect the growth of *T. reesei* NCIM 992 and the production of cellulase were determined (Lee et al. 2011).

### Enzymatic saccharification of sorghum straw

#### Substrate

Sorghum straw was collected from National Research Centre for Sorghum, Hyderabad, India. Dry stem pieces including leaf sheath were processed in a laboratory disintegrator to attain a particle size of 2–3 mm followed by washing with tap water until the washings were clear and then dried at 50 °C for overnight.

#### Delignification

For delignification, the substrate was suspended in 0.2 M NaOH solution in the ratio of 1:10 and kept at room temperature for 18 h. The contents were filtered and the residue was repeatedly washed with tap water until the pH of the residue became neutral. The residue was dried at 50 °C, and subsequently used for acid/enzyme hydrolysis experiments.

#### Acid hydrolysis

The alkali pretreated substrate was subjected to acid hydrolysis with 3% sulfuric acid at 130 °C for 30 min. The contents were filtered and the residue was used for second phase acid hydrolysis experiments.

For enzyme hydrolysis experiments the residue was repeatedly washed with tap water until the pH of the residue became neutral and dried at 50 °C.

#### Biphasic acid hydrolysis

The 3% acid-treated substrates were subjected to second phase acid hydrolysis with 5% sulfuric acid at 130 °C for 45 min. The contents were filtered and the residue was repeatedly washed with tap water until the pH of the residue became neutral. The residue was dried at 50 °C, and subsequently used for enzyme hydrolysis experiments.

#### Enzymatic hydrolysis

The enzymatic hydrolysis experiments were carried out in 100 ml flasks with a working volume of 20 ml.

Untreated (control), alkali treated, alkali treated followed by single phase acid (3%) treated and alkali treated followed by biphasic acid (3 and 5%) treated sorghum straw were hydrolyzed with 25 FPU/g of crude cellulase in 0.05 M citrate buffer (pH 4.8) at a substrate concentration of 5% (w/v). The substrates were soaked in buffer for 2 h before adding the enzyme. Sodium-azide (0.005%) was added to the reaction mixture to prevent microbial or fungal contamination.

The flasks were incubated at 50 °C on an orbital shaker at 150 rpm for 60 h. Sample aliquots of 1 ml were taken at different times, centrifuged and the supernatants were analyzed for reducing sugars to determine the percentage of hydrolysis (% saccharification).

The percentage of hydrolysis was calculated as follows

The delignified sorghum straw after 3% acid hydrolysis when subjected to enzyme hydrolysis showed highest percentage of hydrolysis (42.5%) and hence this substrate was selected for further optimization studies.

### Optimization of enzymatic hydrolysis

Various parameters such as hydrolysis time, temperature, buffer, enzyme loading, substrate concentrations and addition of surfactant, etc., were studied to find out the best hydrolyzing conditions for sorghum straw using cellulase produced by *T. reesei* NCIM 992 on alkali treated rice straw. The optimum condition obtained from each experiment was used in the next optimization study unless otherwise stated.

#### Optimization of hydrolysis time

The enzymatic hydrolysis studies were carried out for a period of 60 h at 50 °C, and we found that maximum hydrolysis (42.5%) occurred at 36 h and remained constant thereafter. Further optimization studies were carried out for 36 h only.

#### Optimization of enzyme loading

The pretreated substrate was loaded with different concentrations of cellulase (10–30 FPU/g) to determine the optimum enzyme concentration required to hydrolyze the substrate. Hydrolysis was performed with 0.05 M citrate buffer (pH 4.8) at 50 °C for 36 h.

#### Optimization of temperature

Hydrolysis was performed at different temperatures (35, 40, 45, 50 and 55 °C) to determine the optimum temperature for the hydrolysis with 25 FPU/g of crude cellulase in 0.05 M citrate buffer (pH 4.8) for 36 h.

#### Optimization of substrate concentration

The substrate was suspended in fixed volume (20 ml) of 0.05 M citrate buffer (pH 4.8), with different weights of substrate (0.2, 0.4, 0.6, 0.8, 1.0 and 1.2 g) and incubated for 36 h at 50 °C.

#### Optimization of surfactant and buffer

Enzyme hydrolysis was performed with 1 g substrate in 20 ml 0.05 M acetate buffer (pH 4.8) at 50 °C for 36 h by the addition of Tween-80 in the range of 0.05, 0.1, 0.15, 0.2 and 0.25 g/g. All experiments were performed in triplicates.

## Results and discussion

### Enzyme production under SSF

Rice straw is a by-product of rice production and a great bioresource. It is one of the abundant lignocellulosic waste materials in the world. Its annual production is about 731 million tons, which is distributed in Africa 20.9 million tons, Asia 667.6 million tons and Europe 3.9 million (Roberto et al. 2003).

According to Kim and Dale (2005) no rice straw must be left on the field to prevent erosion, so that rice straw could be made available for the utilization in bioethanol industry.

Pretreatment (steam, alkali or acid treatment) may reduce the indigenous microflora particularly required in SSF, where key enzymes must be pre-induced for a quick start of lignocellulose breakdown and fungal growth (Tengerdy and Szakacs 2003). Rita Rani et al. (2006) found that pretreatment of substrate increased the cellulase yields by 33%.

Kang et al. (2004) reported 19.5 FPU/g on fourth day by SSF on rice straw using *A. niger* KK2. Our reports on enzyme activity are in close comparison with the findings of Deshpande et al. (2008) who reported 27.39 FPU/g with *T. reesei* (QM 9414 mutant) by SSF using water hyacinth, wheat bran, wood straw and their combinations as substrates.

Furthermore, Rita Rani et al. (2006) reported Fpase activity of 56.5 FPU/gds at 28 °C in SSF using pretreated sugarcane bagasse by *T. reesei* NRRL 11460.

Xu et al. (2008) studied the effect of soluble disaccharides on cellulase activity and found significant enhancement from 11.7 U/ml of Fpase activity to 16.3 U/ml after fifth day when 1% lactose was incorporated in the medium. According to Lee et al. (2011), FPase activity of 3.4 U/g was obtained using local isolate *Aspergillus niger* USM AI 1 grown on sugarcane bagasse and palm kernel cake at 1:1 (w/w) ratio under optimized SSF conditions.

**Fig. 1:**
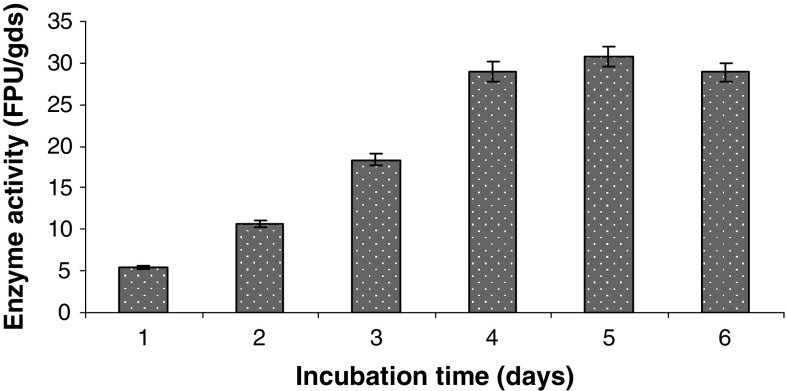
Cellulase production by SSF using rice straw. Data shown are the mean of three experiments (*n* = 3). *Error bars* indicate standard error mean (SEM)

We could obtain (Fig. 1) 30.7 FPU/gds of enzyme activity on fifth day of incubation with 80% moisture level and 1% lactose as inducer at 28 °C under SSF from rice straw using *T. reesei* NCIM 992. The Fpase activity may be further improved by optimizing various parameters and by improving the strain employed.

### Delignification and acid hydrolysis

The holocellulose content of sorghum straw was found to contain (37.51 ± 0.42% cellulose, 26.50 ± 1.5% of hemicellulose, 19.0 ± 0.27% of lignin and 10.02 ± 1.05% of ash) on dry solid (DS) basis which is in accordance with Mehmood et al. (2009). The presence of lignin in cellulosic substrates hinders the saccharification of them into monomeric sugars. Therefore, to overcome the lignin barrier, lignocelluloses are usually pretreated initially with alkali to dissolve the lignin caused by the breakdown of ether linkages (Lee 1997). Efficient delignifying agent should remove a maximum of lignin and minimum of sugars (not more than 5%) (Taherzadeh and Karimi 2007). Chemical alkali pretreatment at ambient temperatures is simple and time-saving and appears to have strong commercial potential (Kim and Holtzapple 2005). Sodium hydroxide is a typical alkali used in alkaline pretreatment (Varga et al. 2002).

Therefore, we have used sodium hydroxide (NaOH) for delignification of sorghum straw for 18 h at 30 ± 2 °C and we could achieve nearly 75% lignin loss with only 3.5% sugar loss.

Acid hydrolysis (3% acid) of the delignified substrate resulted in 45% of hydrolysis and biphasic (3 and 5%) acid hydrolysis of the substrate resulted in 65% (of total cellulose) hydrolysis.

### Enzymatic saccharification of sorghum straw

**Fig. 2:**
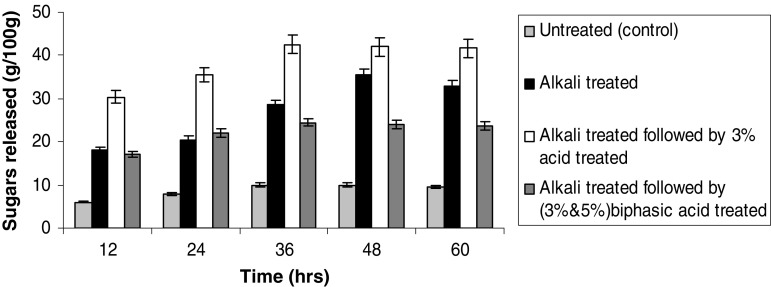
Hydrolysis of differently pretreated sorghum straw with cellulase of *T. reesei*. Data shown are the mean of three experiments (*n* = 3). *Error bars* indicate standard error mean (SEM)

When enzymatic hydrolysis was performed for untreated (control), alkali treated, alkali treated followed by single phase acid (3%) treated and alkali treated followed by biphasic acid (3 and 5%) treated sorghum straw, we observed that alkali delignified followed by acid treated sorghum straw released highest amounts of sugars (42.5%) when compared to other substrates (Fig. 2).

It has been established by Valchev et al. (2009) that the enzyme treatment on raw material as first stage treatment can be inefficient, probably because of impeded access of the enzyme molecules to the amorphous sections of the carbohydrate chains. Furthermore, they have identified considerable improvement in enzyme hydrolysis after acid hydrolysis of crushed maize stalks.

### Optimization of enzymatic saccharification

**Fig. 3:**
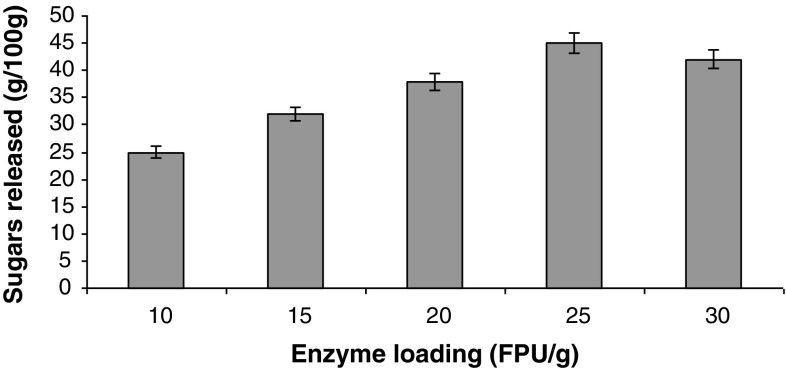
Effect of different enzyme loadings on hydrolysis of sorghum straw. Data shown are the mean of three experiments (*n* = 3). *Error bars* indicate standard error mean (SEM)

For the improvement of enzymatic hydrolysis, it is necessary to optimize the critical process parameters such as optimum cellulase loading, temperature, saccharification time and substrate to liquid ratio. Enzymatic saccharification of the acid pretreated sorghum straw into glucose was optimized using the enzyme supernatant of *T. reesei* NCIM 992. These process parameters play a crucial role in enzymatic hydrolysis of lignocelluloses to get satisfactory yield of monomeric sugars. Optimization of time of enzymatic hydrolysis was done. Figure 2 shows that maximum sugars were released after 36 h showing the hydrolytic efficiency of 331.5 ± 0.5 mg/g sugars with an efficiency of 42.5% at 50 °C. A regular increase in released sugar amount was observed from 12 to 36 h and remained constant thereafter. An optimum enzyme concentration is required to hydrolyze the cellulose into glucose. The effect of enzyme loading on the enzymatic hydrolysis of pretreated sorghum straw has been studied and the results are presented in Fig. 3. The sugar yield increased sharply by increasing enzyme loading from 10 to 25 FPU/g. Thereafter, there was no significant increase in sugar concentration. This shows that an optimum enzyme loading is required to hydrolyze the substrate into glucose. 42.5% of the enzymatic hydrolysis efficiency was achieved at an enzyme loading of 25 FPU/g with our enzyme for sorghum straw. Varga et al. (2002) found that the enzymatic conversion efficiency of corn stover pretreated with NaOH was between 70 and 80% with an enzyme loading of 42 FPU/g.

**Fig. 4:**
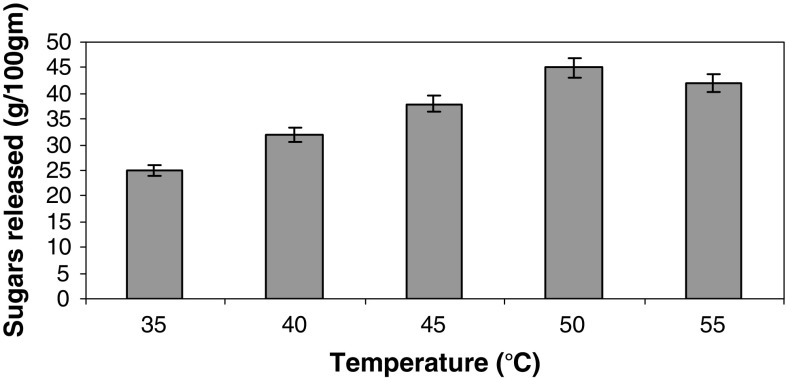
Effect of different temperatures on hydrolysis of sorghum straw. Data shown are the mean of three experiments (*n* = 3). *Error bars* indicate standard error mean (SEM)

The effect of temperature was studied to know the optimum temperature for maximum hydrolysis of cellulose into glucose. Figure 4 shows that maximum sugars released were 351 mg/g of sugars with an efficiency of 42.5% at 50 °C within 36 h. The temperature 50 °C has been used as optimum for enzymatic hydrolysis of different lignocellulosics (Xu et al. 2006). We found similar observations in our studies.

**Fig. 5:**
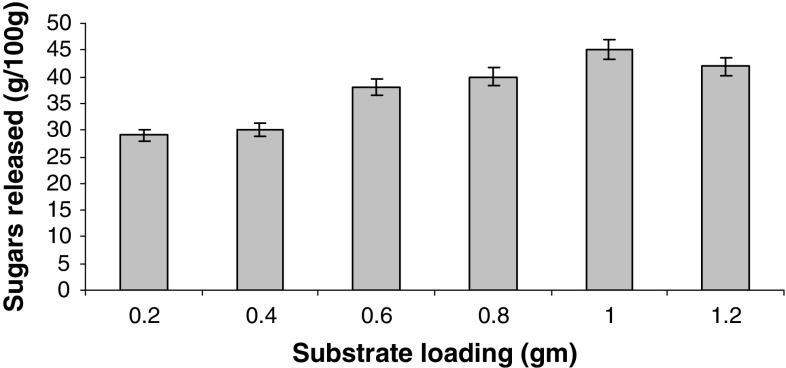
Effect of different substrate loadings on hydrolysis of sorghum straw. Data shown are the mean of three experiments (*n* = 3). *Error bars* indicate standard error mean (SEM)

The ratio of substrate to liquid is also a very important parameter in enzymatic hydrolysis of lignocellulosics. The effect of substrate concentration on enzyme hydrolysis was studied at a fixed ratio of cellulase loading (25 FPU/g) to different concentrations of substrate (0.2, 0.4, 0.6, 0.8, 1.0 and 1.2 g) in a total reaction mixture of 20 ml. As shown in Fig. 5, the rate of conversion decreased when substrate taken was increased from 1 to 1.2 g. Increase in substrate concentration limits the saccharification yields because of stirring difficulties, reduction of aqueous movable phase and end product inhibition (Szczodrak 1999).

**Fig. 6:**
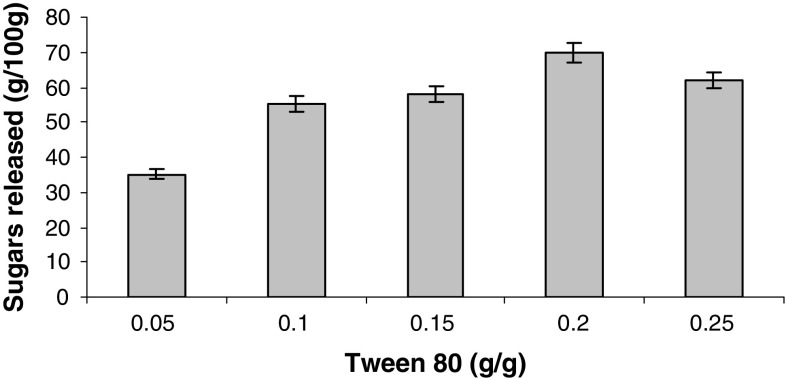
Effect of different concentrations of surfactant on hydrolysis of sorghum straw. Data shown are the mean of three experiments (*n* = 3). *Error bars* indicate standard error mean (SEM)

A maximum of 351 mg/g of sugars with an efficiency of 42.5% at 50 °C after 36 h was obtained. Increasing the substrate level further showed a downfall in sugar amount which clearly indicates that optimum concentration of substrate to buffer is required for maximum hydrolysis of cellulose. The reason could be due to the fact that the rate and extent of biomass hydrolysis are inextricably linked to biomass structural characteristics (Zhu 2005). Optimization of surfactant concentration and effect of buffer on enzyme hydrolysis were also studied when 0.05 mM acetate buffer (pH 4.8) was used for the hydrolysis of sorghum straw with 0.2 g/g of Tween-80. There was an increase in hydrolysis from 45 to 70% (based on cellulosic fraction) with a maximum of 546.00 ± 0.55 mg/g of sugars (Fig. 6).

Recent studies show that enzyme saccharification yields can be enhanced using surfactants (Tween-20, Tween-80 or PEG) or BSA (bovine serum albumin) (Tabka et al. 2006). It has also been reported that surfactants could increase the stability of cellulase (Kaar and Holtzapple 1998). Surfactant as an enzyme stabilizer contributes to better performance of cellulose hydrolysis. Yao et al. (2007) found 20% increase in the enzymatic saccharification of rice straw by the addition of surfactant.

Cara et al. (2008) reported the maximum hemicelluloses recovery (83%) of olive tree biomass to be obtained at 170 °C and 1% sulfuric acid concentration, but the enzyme accessibility of the corresponding pretreated solid was not very high. Saha et al. (2005) found a maximum yield of fermentable sugars (485 mg/g, 64% of total carbohydrates) from acidic pretreated wheat straw [7.83% (w/v), DS, acid dose of 0.75% (v/v)]. Only 47% cellulose (203 mg/gds) was converted to glucose. Recently, Kim et al. (2008) reported the enzymatic digestibility of the ammonia pretreated barley husk to be 83% for glucan and 63% for xylan with 15 FPU/g glucan enzymes loading. Sunflower hulls hydrolyzed with *T. reesei* Rut C30 cellulase (25 FPU/g of substrate) showed 59.8% saccharification after pretreatment with sodium hydroxide 0.5% (w/v) (Sharma et al. 2004). Verônica et al. (2010) obtained 149.6 g/l of glucose from pretreated sugarcane bagasse when 25 FPU/g of substrate was loaded. We could obtain a maximum of 70% (546 ± 0.5 mg/g) hydrolysis of acid treated sorghum straw with crude cellulase of *T. reesei* NCIM 992.

## Conclusion

The present study was aimed at enzymatic hydrolysis of sorghum straw, a lignocellulosic substrate using crude cellulase produced under SSF. Under the optimized SSF conditions, about 30.7 U/g of FPase activity was obtained from *T. reesei* NCIM 992 using KOH pretreated rice straw. When acid treated sorghum straw was subjected to enzymatic hydrolysis with cellulase of *T. reesei* NCIM 992, 70% (546 ± 0.5 mg/g) of sugars were recovered after optimization of enzyme loading, temperature, time and substrate to liquid ratio, addition of surfactant and buffer. In comparison with chemical hydrolysis, enzymatic hydrolysis does not require large volumes of chemicals and usually conducted at mild conditions, hence does not have a corrosion problem. Furthermore, chemical hydrolyzates need to be detoxified before carrying out fermentation. Therefore, enzymatic saccharification for the production of bioethanol from lignocellulosic substrates is an efficient process that has the potential to become most promising method for hydrolysis.

## Abbreviations

Fpase: Filter paperase
PDA: Potato dextrose agar
gds: Gram dry substrate
FPU: Filter paper units
